# Supplementary material for: MicroRNA Expression Profile Changes after Cardiopulmonary Bypass and Ischemia/Reperfusion-Injury in a Porcine Model of Cardioplegic Arrest
Source: Diagnostics (Basel). 2020 Apr 21;10(4):240. doi: 10.3390/diagnostics10040240 (PMC7236010; doi:10.3390/diagnostics10040240)
Supplement: Supplementary file 1 [file diagnostics-10-00240-s001.pdf]

Supplemental Table 1: List of Primer Sequences for Real Time PCR

Gene	Forward	Reverse
Actb	CACGCCATCCTGCGTCTGGA	AGCACCGTGTTGGCGTAGAG
Tnf-alpha	GGCCCAAGGACTCAGATCAT	CTGTCCCTCGGCTTTGACAT
Interleukin-6	ATGTCGAGGCTGTGCAGATT	TTGTGTTCTTCATCCACTCGT
Bcl2	CATGCGGCCTCTATTTGATT	CCCGTGGACTTCACTTATGG
Hmgb1	GGCTGCTAAGCTGAAGGAGA	GCTGCATCAGGCTTCCCTTT
Casp1	GAAGCAAGGGGTCGTATGGA	AGCCCAATAAGGGATGTCGC
Casp3	TTGAGACGGACAGTGGGACT	CGTCCTTTGAATTTCGCCAGG

Supplemental Table 2: Top 20 differently expressed microRNAs identified by next-generation sequencing: on-pump vs. off-pump group.

microRNA ID	logFC	logCPM	p-Value	FDR	on-pump AVG	off-pump AVG
ssc-miR-122	-4.00	5.75	0.00	0.00	6.2	98.3
ssc-miR-10a-5p	-1.28	5.73	0.00	0.00	30.6	74.5
ssc-miR-193a-3p	1.49	3.30	0.00	0.00	14.3	5.0
ssc-miR-499-3p	0.83	6.38	0.00	0.00	106.0	59.5
ssc-miR-374a-5p	0.75	9.96	0.00	0.01	1252.3	744.3
ssc-miR-345-5p	0.99	4.79	0.00	0.01	36.3	18.2
ssc-miR-142-3p	1.05	5.30	0.00	0.01	52.4	25.5
ssc-miR-424-5p	0.79	7.19	0.00	0.01	184.9	106.8
ssc-miR-545-3p	1.13	2.87	0.00	0.02	9.3	4.3
ssc-miR-30b-5p	0.72	11.09	0.00	0.02	2703.3	1641.6
ssc-miR-145-5p	0.66	9.92	0.00	0.02	1183.7	751.5
ssc-miR-374b-5p	0.70	7.44	0.00	0.02	214.9	132.7
ssc-miR-139-3p	-0.77	4.39	0.00	0.02	14.9	25.6
ssc-miR-190a	0.60	7.76	0.00	0.06	259.8	171.9
ssc-miR-758	0.85	3.32	0.00	0.06	12.1	6.8
ssc-miR-130a	0.62	9.37	0.00	0.06	803.1	522.9

ssc-miR-342	0.83	4.29	0.00	0.06	24.6	13.9
ssc-miR-19a	0.55	7.23	0.01	0.06	177.3	121.4
ssc-miR-150	0.67	5.86	0.01	0.06	70.6	44.5
ssc-miR-19b	0.53	10.37	0.01	0.07	1563.1	1084.5

Supplemental Table 3: Top 20 differently expressed microRNAs identified by next-generation sequencing: STH2-cold vs. STH2-warm group.

microRNA ID	logFC	logCPM	p-value	FDR	STH2-cold AVG	STH2-warm AVG
ssc-miR-451	1.50	10.31	0.00	0.00	694	1969
ssc-miR-146b	-0.67	6.28	0.00	0.12	93	59
ssc-miR-144	0.97	9.22	0.00	0.27	416	815
ssc-miR-338	-0.97	5.03	0.01	0.28	42	21
ssc-miR-122	-1.15	2.83	0.01	0.29	9	4
ssc-miR-193a-5p	-0.62	3.11	0.01	0.29	10	7
ssc-miR-20b	-0.55	5.30	0.01	0.40	46	31
ssc-miR-9-1	-0.55	6.58	0.02	0.42	112	76
ssc-miR-195	-0.47	7.77	0.02	0.44	249	180
ssc-miR-9-2	-0.55	6.56	0.02	0.44	110	75
ssc-miR-9	-0.55	5.68	0.02	0.44	60	40
ssc-miR-7137-5p	0.48	3.13	0.03	0.61	7	10
ssc-miR-4334-3p	0.42	5.20	0.04	0.64	32	42
ssc-miR-101	0.36	11.63	0.05	0.74	2812	3617
ssc-miR-542-3p	0.31	9.18	0.05	0.76	523	650
ssc-miR-497	-0.39	9.17	0.06	0.78	646	494
ssc-miR-208b	0.34	14.86	0.06	0.83	26550	33570
ssc-miR-455-3p	-0.37	7.33	0.07	0.84	180	139
ssc-miR-503	-0.36	5.18	0.08	0.85	40	31
ssc-miR-744	-0.40	4.44	0.08	0.85	24	18

Supplemental Table Legends

Supplemental Table 1. List of Primer Sequences for Real Time PCR

Supplemental Table 2. Top 20 differently expressed microRNAs identified by next-generation sequencing: on-pump vs. off-pump. The p-value describes the significance level for the fold change. The adjusted p-value describes the significance level after adjusting for multiple testing using Benjamini-Hochbergs method for false-discovery rate (FDR).

Supplemental Table 3. Top 20 differently expressed microRNAs identified by next-generation sequencing: STH2-cold vs. STH2-warm group. The p-value describes the significance level for the fold change. The adjusted p-value describes the significance level after adjusting for multiple testing using Benjamini-Hochbergs method for false-discovery rate (FDR).
